# Influence of Heat Stress on Body Temperatures Measured by Infrared Thermography, Blood Metabolic Parameters and Its Correlation in Sheep

**DOI:** 10.3390/metabo13080957

**Published:** 2023-08-18

**Authors:** Aleksandar Čukić, Simeon Rakonjac, Radojica Djoković, Marko Cincović, Snežana Bogosavljević-Bošković, Milun Petrović, Željko Savić, Ljiljana Andjušić, Biljana Andjelić

**Affiliations:** 1 Faculty of Agriculture, University of Priština in Kosovska Mitrovica, Kopaonička bb, 38219 Lešak, Serbia; aleksandar.cukic@pr.ac.rs (A.Č.); zeljko.savic@pr.ac.rs (Ž.S.); ljiljana.andjusic@pr.ac.rs (L.A.); 2 Faculty of Agronomy, University of Kragujevac, Cara Dušana 34, 32000 Čačak, Serbia; simeonr@kg.ac.rs (S.R.); sbb@kg.ac.rs (S.B.-B.); milunp@kg.ac.rs (M.P.); 3 Faculty of Agriculture, University of Novi Sad, Novi Sad, Square Dositeja Obradovića 8, 21000 Novi Sad, Serbia; mcincovic@gmail.com; 4 Faculty of Agriculture-Kruševac, University of Niš, Kosančićeva 7, 37000 Kruševac, Serbia; andjelic.biljana@ni.ac.rs

**Keywords:** sheep, heat stress, blood parameters, body temperature, thermoregulation, adaptation

## Abstract

The aim of this research is to examine the influence of heat stress (HS) on body temperature (BT) measured rectally (RT) or by infrared thermography (IRT) of the nose (NT), eye (ET), leg (LT) and abdominal (AT) regions in intensively and extensively breed sheep and to detect a correlation between body temperature and metabolic response in sheep. A total of 33 Wurttemberg × Sjenica Pramenka sheep breeds were examined, 17 ewes were from outdoors and 16 were from indoor housing systems during three experimental periods (thermoneutral period, severe HS and moderate HS). Sheep under HS have a higher BT, and the magnitude of BT measured by infrared thermography (IRT) was higher than RT. LT and AT showed positive linear correlations with the temperature–humidity index (THI), while other ways of measuring BT did not give statistically significant correlations. Sheep under HS showed higher cortisol, insulin, total protein, albumin, urea, creatinine, bilirubin, aspartate aminotransferase, alanine aminotransferase, gamma-glutamyl transferase, alkaline phosphatase, lactate dehydrogenase, creatine kinase and index of insulin resistance, with lower values of triiodothyronine (T3), thyroxine (T4), non-esterified fatty acids, beta-hydroxybutyrate (BHB), glucose, calcium, inorganic phosphates, magnesium and cholesterol. BT and metabolic response were different in the function of the housing method of sheep. LT and AT showed a significant correlation with almost all blood parameters, and the strongest connections were made with T3, T4, BHB and the revised quantitative insulin sensitivity check index of insulin resistance. The abdomen and legs are good thermal windows because LT and AT are good summative responses to external ambient THI and internal metabolic changes in sheep under heat stress.

## 1. Introduction

Global warming is a significant risk to animal and human health, as it affects the living world from various aspects. Heat stress is one of the most harmful factors that contribute to reduced growth, production, reproduction, quantity and quality of milk, as well as natural immunity, making animals more vulnerable to disease and even death [1]. However, small ruminants have successfully adapted to this extreme environment and possess some unique adaptive traits based on behavioural, morphological, physiological and above all genetic principles. Complete information is lacking on how these animals can adapt and survive in new and changing environments [1]. In general, sheep have good adaptability, and they are resistant to harsh environmental conditions. However, physiological and behavioral changes in response to hot environments affect the production of small ruminants [2]. 

In most countries of the Balkan Peninsula, including Serbia, sheep are raised in extensive farming systems, with a small percentage of intensive sheep farming. Much of the production takes place on extensive pastures where inputs are low, and fodder production and water supply vary according to the seasonal climate. The impact of heat stress on welfare and health is much more pronounced in high-production ruminants, both in intensive and extensive husbandry [3]. The adaptive profiles of sheep have been well-studied in different farming systems [4,5]. The degree to which these stress impacts on productivity will differ between the agroecological regions and between production systems [6]. Temperature and humidity index (THI) is the simplest approach, which combines ambient temperature and relative humidity to assess the response of livestock productivity as a function of climate [7]. As temperature and humidity are often readily collected, the minimal inputs make THI an easy tool for retrospective studies in most regions [8].

Body temperature is an important indicator of the health status of animals. In practice, different methods of temperature measurement are used. Rectal temperature generally gives a good indication of body core temperature. The greater magnitude of rectal temperature increase in small ruminants during periods of heat stress suggests that these animals can store body heat during periods of heat stress [9]. Measuring the heat emitted by the body is becoming more and more popular, and the thermal imaging camera has found its wide application. The thermal imaging camera represents a modern and non-invasive assessment of thermal status. The principle of operation of the infrared camera is based on measuring the radiation emission in the object or on a certain part of the animal body and recording the measured temperature in the form of an image [10]. Thermo-graphic images can be used to demonstrate an increase in body temperature and changes in blood flow related to stressful environmental conditions such as high heat load [11]. Due to its non-obstructive nature, this technology is well-suited for the assessment of stress and welfare [12] and has been used as a diagnostic tool to predict heat stress [13]. Additionally, studies have assessed the use of IRT in livestock to determine heat tolerance [14] and thermal thresholds [15] and to predict the effects of heat stress on reproductive output [16]. 

Exposure to elevated ambient temperature evokes a series of drastic changes in the sheep’s biological functions that include depression in feed intake efficiency and utilization, disturbances in the metabolism of water, protein, energy and mineral balances, enzymatic reactions, hormonal secretions and blood metabolites [17]. Performance strongly affected by high temperatures includes a range of neuroendocrinological, physiological and behavioral responses, which affect the balance of animal functions [18]. Such responses can promote alterations in the level of blood metabolites and metabolic hormones such as T3, T4 or insulin [17,19,20]. The thermoneutral zone for sheep is generally between 12 and 27 °C [18,21]. Activation of compensatory and adaptive mechanisms allows sheep to efficiently tolerate temperatures above the upper limit of their thermoneutral zone without drastically compromising their productivity [22]. In the current perspective of global climate change, it is essential to understand the effect of environmental changes on the organism as well as the adaptive mechanisms in their arsenal to combat them [6].

We hypothesized that body surface heat measured with a thermal imaging camera is the result of external environmental factors and internal metabolic response during heat stress in sheep. The aim of this research was to examine the influence of heat stress (HS) on metabolic response and body temperature (BT) measured rectally (RT) or by infrared thermography (IRT) of the nose (NT), eye (ET), leg (LT) and abdominal (AT) regions in indoor and outdoor housing sheep and to detect a correlation between body temperature and metabolic response in sheep.

## 2. Materials and Methods

### 2.1. Animals and Management 

The experiment was conducted in Serbia, location Lešak (43°09′59.7″ N 20°44′35.5″ E, altitude 455 m), in April, June and July 2022 in two different farms. One farm is engaged outdoors, and the other is in indoor sheep housing, i.e., pasture and stable breeding of sheep. A total of 33 Wurttemberg x Sjenica Pramenka sheep breeds were examined, 17 ewes were from the outdoor housing farm, and 16 were from the indoor housing farm. In outdoor housing, sheep spend most of the year grazing, except from December to March when they are in facilities. Therefore, the animals move freely in huge spaces, more or less covered with grass (from 3 to over 7 cm sward height), so the diet is exclusively based on pasture, while in the cold period of the year, on hay and small amounts of concentrated nutrients, they are given water 2 to 3 times a day. In indoor housing, sheep are kept in the barn all year round, where food is brought to them. The diet is based on silage ad libitum and concentrates in the amount of 250–300 g per sheep per day, and watering is done ad libitum with automatic watering cans. Animals are in lactation and are 2–3 years old. Silage is a mix of maize and hay silage with 320–370 g/kg of dry matter (DM), including the following components of DM(g/kg) during the year: organic matter 920–930, crude protein 88–100, neutral detergent fiber 680–705 and acid-detergent fiber 409–419 and metabolic energy 9–12 MJ/kg DM. Concentrate was based on barley, maize, sugar beet pulp, rapeseed meal, soya bean meal, molasses, and minerals with the following chemical characteristics: dry matter 860–880 g/kg, ME 12–13 (MJ/kg DM), effective rumen degradable protein 112–125 (g/kg DM) and Digestible Undegradable Protein 31–37 (g/kg DM). Sheep were sheared in April, before the first period of the experiment. The body weight of the sheep was uniform and averaged 59.5 ± 2.1 kg, with normal body condition and without fat or thin sheep. Sheep are not used for milking, so there are no data on milk production. Ventilation in the barn is done naturally by opening doors and windows. The area for sheep is 60 m^2^ per sheep, 1.6 m^2^, with the height of a barn of 3 m, which is in accordance with the standards [23]. This is a monoslope build where the entire roof slopes in one direction. Summer ventilation is accomplished using a cross-flow of air from one sidewall to the other, while winter ventilation is accomplished by air entry and discharge through openings in the highest wall. Only healthy animals with healthy hoofs, uniform breed, structure, and hair length and without signs of disturbance participated in this experiment.

Sheep restraint—the restraint of the sheep was carried out using the headstand construction, which allowed the head to be supported and fixed, after which the sheep stood still. The animal’s body may also need to be restrained. The head was raised and allowed an easy finding of the vein. The same rack was used for all sheep. An electric shear is used to shave a patch approximately 10 cm wide by 20 cm long. The easiest way to locate the vein is to draw an imaginary line from the middle of the animal’s eye down the side of its neck. The vein is located by applying pressure with the thumb or fingers below the halfway point of the shaved area. The pressure will cause the vein to pop up and be easy to see. Once the vein has been located, the area needs to be properly cleaned to keep bacteria out of the needle insertion site. This is accomplished by using the alcohol spray on the area. Never go back over a place that has already been wiped because bacteria could be carried back into the clean area. The collection site is cleaned with three alternating scrubs of 70% alcohol and betadine. Infrared imaging, blood sampling and rectal temperature were measured during one capture of the sheep. The whole procedure took a few minutes per sheep.

### 2.2. Body Temperature and Ambient Temperature–Humidity Index

For the purposes of calculating THI, temperature and air humidity were measured three times a day in three different periods of the experiment, at 07:00 (THI morning), 14:00 (THI day) and 19:00 (THI night). The temperature–humidity index was calculated using the standard formula [8] according to data from the Republic Hydrometeorological Service of Serbia and data obtained from our weather station (Sencor SWS 51 B). 

Rectal temperature was measured by a standard digital thermometer. The body surface temperature was measured in all animals by collecting images in different parts of the body-thermal window: eye temperature (ET), nose (NT), front leg (LT) and abdomen temperature (AT). Thermographic images were obtained with an infrared camera, Testo 865 (Titisee-Neustadt, Germany). Thermograms with an emissivity coefficient of 0.95 were recorded from each sheep. Thermal images of the eyes and nose were obtained at a distance of approximately 20 cm, and the front leg and the abdomen images at a distance of less than 1 m. Thermal imaging was performed in three different periods of the experiment, in a time interval of 12:00–16:00 h, simultaneously with blood sampling.

### 2.3. Blood Sampling and Metabolic Parameters Analysis

Blood samples were collected by jugular venipuncture using 10 mL serum separation tubes. In order to separate the serum better, it was additionally centrifuged for 5 min at 3000× *g*. The serum samples were then collected and placed in vials, frozen at −20 °C and transported by laboratory refrigerators to the Laboratory of Pathophysiology, Department of Veterinary Medicine, University of Novi Sad. We performed analysis for the following biochemical parameters: cortisol (CORT), triiodothyronine T3, thyroxine T4, insulin (INS), non-esterified fatty acids (NEFA), beta-hydroxybutyrate (BHB), glucose (GLU), calcium (Ca), inorganic phosphates (P), magnesium (Mg), total protein (TPROT), albumin (ALB), globulin (GLB), urea, triglycerides (TGC), cholesterol (CHOL), total bilirubin (TBIL), direct bilirubin (DBIL), indirect bilirubin (IBIL), creatinine (CR), aspartate aminotransferase (AST), alanine aminotransferase (ALT), gamma-glutamyl transferase (GGT), lactate dehydrogenase (LDH), alkaline phosphatase (ALP) and creatine kinase (CK). Standard kits from Randox (Crumlin, UK) for NEFA and BioSystem (Barcelona, Spain) for other parameters were used on Rayto Chemray 120 spectrophotometer (Rayto Life and Analytical Sciences, Shenzhen, China). An automated immunoassay analyzer, TOSOH AIA-360 (Tosoh Bioscience, Tokyo, Japan), was used for endocrinological analyses. For the estimation of insulin sensitivity, we used the homeostasis model assessment (HOMA), the quantitative insulin sensitivity check index (QUICKI), the revised quantitative insulin sensitivity check index (RQUICKI), and its modified version (RQUICKI_BHB_). The following formulas [24] were used for calculating these indicators: HOMA = glucose (mmol/L) × insulin (µU/mL)/22.5, QUICKI = 1/[log (glucose mg/dl) + log (insulin μU/mL)], RQUICKI = 1/[log (glucose mg/dL) + log (insulin μU/mL) + log (NEFA mmol/L)] and RQUICKIBHB = 1/[log (glucose mg/dL) + log (insulin μU/mL) + log (NEFA mmol/L) + log (BHB mmol/L)]. 

### 2.4. Statistical Analysis

Statistical analysis included the use of GLM and ANOVA analysis to examine the effect of period, breeding method and period × housing method interaction on the value of body temperature measured rectally or by thermography of different body parts and blood biochemical parameters. The main findings during thermography of the eye, nose, front leg and abdomen in intensive and extensive breeding in all three measurement periods are graphically presented. The connection between thermography and the THI index was examined, as well as the connection between thermography and metabolic parameters using the Pearson correlation coefficient. SPSS statistics software (IBM, Armonk, NY, USA) was used. All statistical tests were considered significant if *p* < 0.05. 

## 3. Results

### 3.1. Temperature–Humidity Index (THI) and Environmental Stress

**Figure 1:**
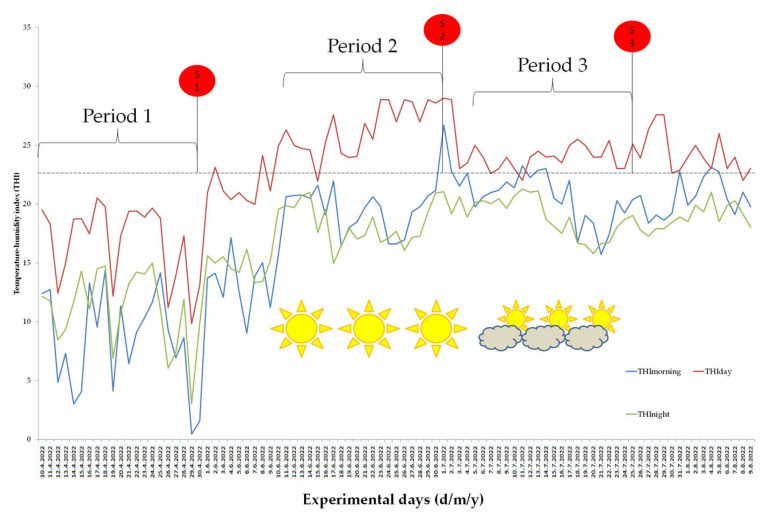
Temperature–humidity index in experimental months (April, June, July) with moment of sampling in Periods 1, 2 and 3.

The results of the research show that the average value of THI measured during the day indicated no HS in Period 1. In Period 2, there is a moderate to strong HS, while in Period 3, there is a weak to moderate HS. The reason for such a finding is the onset of the rainy period, which reduced the ambient temperature in Period 3. The average daily THI value indicates HS, while the THI during the evening and morning hours is not outside the thermoneutral range. The THI value changes over time in an identical manner in indoor and outdoor housing, with the fact that it is, on average, higher by 0.22 units in intensive breeding. This difference does not affect the stress load and the interpretation of heat stress, so we opted for the average presentation of THI results over time. The obtained results are shown in Figure 1.

### 3.2. Body Temperature Measurement

Body temperature (BT) measured rectally or by thermography of the nose, eye, leg or abdominal region significantly differs in function of the examined period, breeding method and their interaction. A significantly higher BT (both rectal and infrared) was measured in the period of strong heat stress (Period 2) compared with other periods (significant effect of the period). A higher RT is found in sheep under indoor housing, but the temperature obtained by IRT is significantly higher in sheep under an extensive breeding system (significant effect of breeding). The change in BT in sheep in extensive breeding during heat stress is much more intense than in sheep in intensive breeding, so the period × breeding interaction is significant. The results are shown in Table 1.

**Figure 2:**
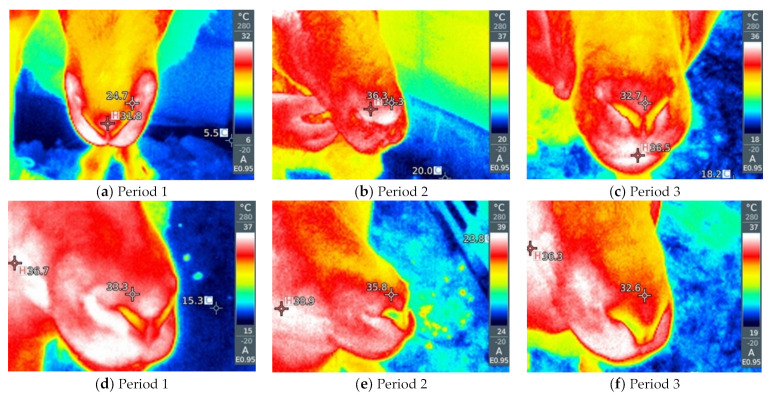
(**a**–**f**) Infrared thermography of the nose, (**a**–**c**) on pasture and (**d**–**f**) in the barn in three exp.

**Figure 3:**
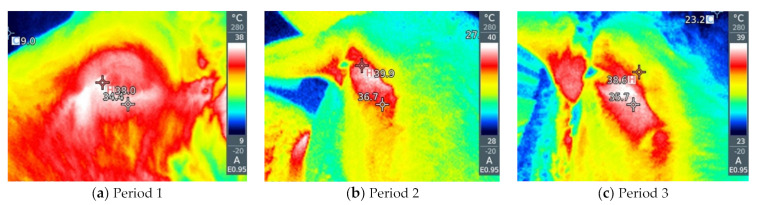

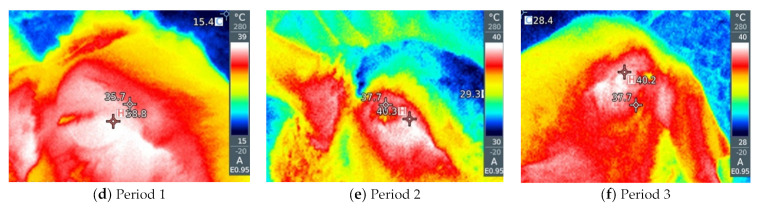
(**a**–**f**) Infrared thermography of the eye, (**a**–**c**) on pasture and (**d**–**f**) in the barn.

**Figure 4:**
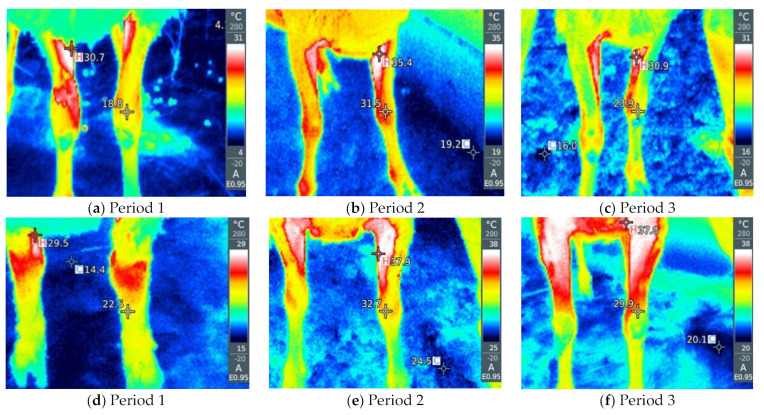
(**a**–**f**) Infrared thermography of the front leg, (**a**–**c**) on pasture and (**d**–**f**) in the barn.

**Figure 5:**
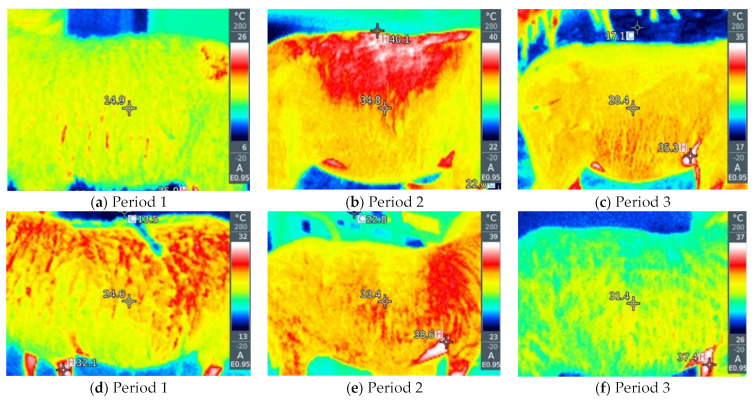
(**a**–**f**) Infrared thermography of the abdomen, (**a**–**c**) on pasture and (**d**–**f**) in the barn.

**Figure 6:**
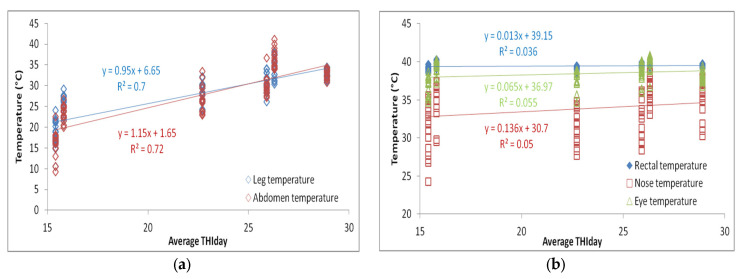
Linear correlation and regression between average daily THI and (**a**) leg and abdomen temperature and (**b**) rectal, nose and eye temperature.

By visual analysis of the infrared thermograph, we conclude that thermographic methods are much more sensitive to changes in ambient conditions compared with rectal temperatures. RT gives a small value deviation between the groups as a function of the period and way of breeding the sheep (by 0.1–0.6 °C). Slightly larger deviations are given by NT and ET between different groups (by about 2–5 °C). LT and AT give the highest deviations between sheep in the thermoneutral period and during HS (by 16–20 °C). Within thermographic methods, the most sensitive are LT and AT, which is also very important for the visual assessment of the obtained thermograph, where it is important that the differences be more noticeable. The obtained results for all four thermographic methods are shown in Figure 2, Figure 3, Figure 4 and Figure 5. A significant positive correlation is found between THI with LT and AT (*p* < 0.001) (Figure 6a), but there is no significant linear correlation of THI with RT, NT and ET (Figure 6b).

### 3.3. Blood Metabolic Parameters

HS significantly affects the values of all examined biochemical parameters, except for TGC, HOMA and QUICKI index. HS in sheep leads to significant metabolic changes characterized by increased values of CORT, INS, TPROT, ALB, urea, CR, all bilirubin, AST, ALT, GGT, ALP, LDH, CK, RQUICKI and RQUICKIBHB index, while a decrease in the values of T3, T4, NEFA, BHB, GLU, Ca, P, Mg and CHOL is found. The breeding method has a significant effect on the values of CORT, INS, Ca, Mg, ALB and urea, which are higher in the extensive group, and T3, T4, GLU, TGC and CHOL, which are lower in the extensive group. Period × breeding method interactions are not statistically significant, except for the value of CR. The results are shown in Table 2.

### 3.4. Correlation between Blood Parameters and Body Temperature

The correlation of blood biochemical parameters and RT is not statistically significant, except for CHOL and TGC. NT and ET show a statistically significant but weak linear correlation with certain biochemical blood parameters. LT and AT values show a statistically significant linear correlation with almost all blood parameters. The strength of correlation of these two methods of thermography with biochemical parameters ranges from weak (CHOL and all bilirubin, P, AST, GGT) to moderate (CORT, INS, NEFA, BHB, GLU, Ca, Mg, TPROT, ALB, urea, CR, ALT, ALP, LDH, CK, RQUICKI, RQUICKIBHB) and strong (T3, T4). The results are shown in Table 3.

## 4. Discussion

THI was used to assess the level of HS, and the results showed an increase in THI during the summer months, which is consistent with the findings of many previous studies. In the study by Hossein-Zadeh [25], it was found that the highest values of the THI were recorded in June, July, August and September. Similar data were recorded in other countries [26,27,28]. Period 1 proved to be a thermoneutral period, in which there is a narrow range of ambient temperatures that are favorable for normal production and welfare, which is at a THI value below 22.2. Already at the beginning of summer, the results showed that the THI values exceeded the upper critical point, in which HS begins to adversely affect the animals, which is above 22.2 THI values in Periods 2 and 3. Based on THI values, HS in Periods 2 and 3 existed during the day, which is expected, while during the evening and morning hours, it did not. Similar to this study, McManus et al. [14] found the highest values of THI in the afternoon, also in the research of Neves et al. [29] and Paim et al. [30] showed that animals suffered the highest thermal stress at 12 h. Finocchiaro et al. [31] found that HS affects the production of Mediterranean dairy sheep at THI ≥ 23, while Sevi et al. [32] reported that HS affects Comisana sheep when THI ≥ 27. Although sheep are more resistant to HS than larger ruminants, based on the findings, we conclude that THI above 23 has a harmful effect on sheep. Data on t and RH obtained with the help of weather station Sencor, in comparison with data on t and RH from the Republic Hydrometeorological Service of Serbia, do not show significant differences, which means that home weather stations can be used on farms to assess weather conditions.

When an animal is stressed, the hypothalamic–pituitary–adrenocortical axis is activated, and heat is produced as a result of increased catecholamine and cortisol concentrations, leading to changes in heat production and heat loss from the animal [33,34]. Thermographic images can indicate changes in blood flow resulting from increased body temperature associated with stressful environmental conditions [30]. It is important that IRT is performed at different sites because the use of a single surface temperature to identify thermal zones is questionable due to differences in vasoconstriction/vasodilatation activity between different parts of the body [35]. Different parts of the body, better known as thermal windows, show a direct connection with the autonomic nervous system, so infrared heat is dissipated there [36,37]. These are the regions that we also used in our experiment, such as the orbital region and the nasal region. In addition to this, we also determined the temperatures of the abdomen and extremities because they are under the additional influence of the ambient environment (sunlight and the floor). 

As for the rectal temperature, it is marked as a parameter that is widely used to determine the degree of adaptability of animals because an increase in this variable indicates that the animal accumulates heat so that thermal stress can be manifested, the normal RT in sheep varies from 38.5 to 39.9 °C [20]. RT did not significantly change in relation to the period and method of breeding sheep, and it was higher than IRT, which is in agreement with the results reported by McManus et al. [14] in lambs, Martello et al. [38] studying feedlot-raised cattle and Berry et al. [39] in dairy cattle. To determine HS in sheep, Starling et al. [40] found that assessment of response to HS using RT was insufficient. The results showed that the body surface temperature (BST) is influenced by the period and the breeding method of sheep. Among the studied parts of the body, in our study, IRT of the abdomen and front legs show the greatest deviations between the period and the breeding method, so it was shown that there is a significant positive correlation between these parts of the body and THI, where AT and LT were much higher in relation to on NT and ET under high and moderate THI conditions, indicating that these body parts may be the most suitable location for HS prediction by thermography. It is interesting that in the thermoneutral period, sheep intensively kept show a higher temperature compared with sheep that were extensively kept, which means that when the temperatures are pleasant, the sheep in the pasture are in a better health condition than the sheep in the barn, while in Period 2, in which the highest THI was measured, intensively kept sheep show lower temperatures, which we conclude is due to the shelter they have from the direct sunlight to which grazing sheep are exposed. In the study by Paim et al. [41] forty lambs were exposed to three different climatic conditions: outdoor, housing and artificial heating, where it was shown that the BST tended to increase as the thermal comfort index increased, so the temperatures of all studied body parts differed in according to the breeding method. NT and ET had a different pattern compared with other temperature points, while in our study, NT and ET did not show a significant correlation with THI. In dairy cattle, in a study by Salles et al. [42] and Peng et al. [43], there were no significant correlations between THI and RT and ET, while there were between THI and AT and LT, which is similar to our findings. Also, Peng et al. [43] concluded that all body parts studied had a higher correlation with THI than RT, suggesting that BST are more sensitive to the thermal environment than RT. The fact that the correlation between THI, AT and LT is much higher than other parts of the body indicates that these regions have the potential to indicate possible influences of the climatic environment on the thermoregulatory responses of sheep.

The effects of HS on blood metabolite concentrations widely vary across studies, making it difficult to explain the metabolic response made by sheep to survive and adapt to hot climates. Factors such as breeding method, period, breed, diet, physiological status and others must be taken into account when interpreting the results. The concentrations of CORT, INS, TPROT, ALB, urea, CR, all bilirubin, all enzymes, RQUICKI and RQUICKIBHB index in HS conditions were higher than in the thermoneutral period. In contrast, the activities of T3, T4, NEFA, BHB, GLU, Ca, P, Mg and CHOL were lower under HS conditions. HS did not affect the values of the TGC, HOMA and QUICKI index. Endocrine responses are one of the main regulators of animal response to HS challenges. Cortisol, the main glucocorticoid, is mainly produced in the adrenal cortex, is considered an important stress marker, and participates in various bodily functions, including immune responses and protein, carbohydrate, and fat metabolism [44]. Higher temperature and THI during summer cause HS, and in response, CORT levels may increase [45,46], so blood CORT concentrations in animals can provide information about their stress status. Under conditions of HS, it is common to observe a decrease in the levels of thyroid hormones T3 and T4, both responsible for mediating animal metabolism, as a mechanism to reduce metabolic heat production [47]. Decreased concentrations of circulating T3 and T4 have been reported, indicating an attempt to decrease metabolic rate and, thus, metabolic heat production in sheep [48]. Insulin is a metabolic hormone important in the regulation of energy metabolism under conditions of HS in sheep [49]. Thus, while HS reduces food intake, hyperinsulinemia prevents lipolysis and increased NEFA concentrations, an excess of which can cause pancreatic β-cell apoptosis [50]. The main indicators of lipomobilization in blood in ruminants are BHB, the most important and abundant ketone body, and NEFA, whose activities in our study were reduced under conditions of HS. Low concentrations of NEFA are mainly reported in dairy cows under HS. This is thought to be an attempt to increase glucose utilization which will result in less metabolic heat production [51,52]. Regarding the index of insulin resistance, HS did not affect the values of the HOMA and RQUICKI index, while it increased the values of the RQUICKI and RQUICKIBHB index. The concentration of total proteins increases in HS. Similar to our findings, Salem et al. [53] observed that serum TPROT levels were higher during hot summer than winter in Chios lambs and crossbred Chios × Ossimi in Upper Egypt, and ALB levels were higher compared with GLB. GLB levels were insignificantly altered by season (winter, summer, autumn) in Ossimi × Suffolk rams under Egyptian conditions [54]. HS is known to induce peripheral vasodilation to expel body heat and reduce blood flow to internal organs [55]. The increase in serum urea and CR in sheep due to HS may indicate that their kidneys experience reduced blood flow during the HS condition. In the current study, we found the same tendency. Indicators of the functional state of the liver are the concentration of bilirubin and the activity of enzymes in the blood, which can also indicate metabolic stress. The values of bilirubin and all enzymes were increased in conditions of HS. In the research of Badakhshan et al. [56], the sensitivity of sheep to HS was shown by an increase in TBIL, indicating signs of red blood cell rupture or liver damage. AST and ALT are two important metabolic enzymes that increase during exposure to HS in sheep [57]. The increase in ALT and AST in the hot period is consistent with the findings of Srikandakumar et al. [55] and Rathwa et al. [58]. The increase in ALT and AST may be due to an increase in gluconeogenesis or due to some adverse effect of HS on liver activity [58,59]. In the study by Hrkovic-Porobija et al. [60] carried out on 117 Pramenka sheep during the summer, the levels of serum enzymes ALT, AST, GGT, ALP and LDH were examined. An increase in all examined enzymes was observed, and the authors concluded that this increase may indicate intensive metabolic processes as a response of the liver to a negative energy balance. These findings are in accordance with our results, and, also in our study, a higher CK activity was observed, which indicates muscle damage, fatigue and susceptibility to HS. HS significantly changes glucose homeostasis in animals. The results of the effect of high ambient temperature on the blood glucose content of sheep are conflicting. In our study, the effect of HS reduced blood GLU value, Marai et al. [54] found in Ossimi sheep that blood GLU levels were significantly higher in summer than in winter. Some other studies showed that blood GLU significantly decreased with different percentages (in Chios sheep and crossbred Chios × Ossimi) [53]. Srikandakumar et al. [55] examined the effect of HS on the metabolism of Omani and Merino sheep, where they observed that HS increased GLU in Merino but decreased it in Omani sheep. HS negatively affects the balance of minerals in the blood, so in our study, it reduced the levels of Ca, P and Mg. Srikandakumar et al. [55] reported a decrease in blood Ca in Omani and Merino sheep, and Baumgarther and Perthanner [61] observed that the level of inorganic phosphorus was significantly lower in summer than in winter in Karakul sheep. Concentrations of Ca, P and Mg were lower under HS conditions in Iranian fat-tailed sheep [57], which the authors concluded to be a consequence of possible reduced food intake associated with HS. Serum total lipid concentration significantly decreases in ruminants with prolonged exposure to high ambient temperature, especially cholesterol values [62,63]. This phenomenon may be the result of an increase in water content in the body or the use of fatty acids for energy production as a result of a decrease in GLU concentration. A marked increase in CORT levels in animals under HS may be another factor that causes a decrease in blood CHOL [17]. Similar to our findings, a study by Macías-Cruz et al. [64] found that HS alters CHOL but not TGC concentrations in sheep. 

The breeding method had an impact on the values of many parameters, where the concentrations of CORT, INS, Ca, Mg, ALB and urea were higher in sheep that were reared extensively, while the values of T3, T4, GLU, TGC and CHOL were higher in sheep in an intensive breeding system (*p* < 0.05 or *p* < 0.01 for all parameters). Many authors confirm that the breeding method affects the biochemical parameters of the blood in conditions of HS, so the study by Karthik et al. [65] in Nellore sheep with different husbandry systems showed higher levels of GLU, TPROT, ALB, CHOL, T3, T4, Ca and P in the intensive system; however, GLB, CR, AST, ALT, glutathione peroxidase and catalases were elevated in extensive and semi-intensive systems. It has been shown that HS affects the value of almost all investigated parameters of blood biochemistry, i.e., energy, protein and mineral status, as well as parameters of indicators of the functional state of the liver, all of which can be used as an important indicator of HS.

The correlation of examined blood parameters and body temperature measured by a digital thermometer and thermography significantly differs between different parts of the body. RT did not correlate with blood biochemical parameters, except for CHOL and TGC. NT and ET showed a weak correlation with certain parameters, while LT and AT showed a significant correlation with almost all blood parameters. This was the first study that monitored the correlations between blood biochemical parameters and IRT under conditions of HS in sheep. Some studies have linked ET measured by IRT to CORT levels in cattle and pigs as a result of altered blood flow induced in this region in response to stressful conditions [66,67], which does not appear to have occurred in our study. IRT is a good indicator of metabolic phenotype in mice [68]. Additionally, IRT is a good method to predict rectal temperature [69], and if we add to that the connection with a metabolic response, we can see the advantage of using this method in everyday practice.

## 5. Conclusions

Under the influence of heat stress, body temperature increases in sheep, and this increase is more pronounced in extensively reared animals. Metabolic changes caused by heat stress are equally pronounced in sheep in intensive and extensive breeding. The use of a thermal imaging camera in the assessment of heat load in sheep shows great advantages over rectal temperature measurement due to its sensitivity to ambient conditions. The temperature of the abdomen and front legs significantly correlates with the THI value but also with the values of metabolic parameters, so it could be a suitable method in the non-invasive assessment of stress load in sheep. This could find its application, especially in extensive sheep breeding. LT and AT are good summative responses to external ambient THI and internal metabolic processes in sheep under heat stress.

## Figures and Tables

**Table 1:** Effect of heat stress and housing method on thermographic data.

Temperature	Period 1		Period 2		Period 3		Period	Housing	Period × Housing
	Ext	Int	Ext	Int	Ext	Int			
Rectal temp. (RT)	39.1 ± 0.35 ^A^	39.7 ± 0.35 ^B^	39.6 ± 0.21 ^B^	39.5 ± 0.21 ^B^	39.2 ± 0.26 ^A^	39.5 ± 0.23 ^B^	0.017	0.000	0.000
Nose temp. (NT)	31.3 ± 3.32 ^A^	35.3 ± 2.5 ^B^	36.5 ± 1.71 ^C^	34.8 ± 2.46 ^B^	31.6 ± 2.42 ^A^	33.1 ± 2.6 ^B^	0.000	0.017	0.000
Eye temp. (ET)	36.8 ± 1.3 ^A^	39.3 ± 0.91 ^B^	39.8 ± 1 ^B^	38.1 ± 0.71 ^C^	37.8 ± 1.19 ^C^	38.7 ± 1.18 ^B^	0.002	0.014	0.000
Leg temp. (LT)	18.3 ± 3.26 ^A^	24.7 ± 2.61 ^B^	34.2 ± 2.33 ^C^	32.7 ± 2 ^D^	28.1 ± 2.44 ^E^	30.6 ± 2.26 ^F^	0.000	0.000	0.000
Abdomen temp. (AT)	16.01 ± 3 ^A^	23.4 ± 1.84 ^B^	36.4 ± 2.21 ^C^	32.7 ± 1.09 ^D^	26.9 ± 3 ^E^	30.1 ± 2.19 ^F^	0.000	0.000	0.000

^A,B,C,D,E,F^—different superscripts indicate statistically significant differences between subgroups.

**Table 2:** The influence of heat stress and breeding method on metabolic parameters.

Temperature	Period 1		Period 2		Period 3		Period	Breeding	Period × Breeding
	Ext	Int	Ext	Int	Ext	Int			
CORT * (nmol/L)	77.9 ± 25.1 ^A^	48.14 ± 17 ^B^	117.5 ± 14 ^C^	97.6 ± 11.8 ^D^	95.8 ± 22.1 ^D^	77.7 ± 11.2 ^A^	0.000	0.000	0.352
T3 (nmol/L)	1.43 ± 0.32 ^A^	1.53 ± 0.3 ^A^	0.51 ± 0.1 ^B^	0.62 ± 0.1 ^C^	0.76 ± 0.2 ^C^	0.89 ± 0.2 ^D^	0.000	0.012	0.957
T4 (nmol/L)	41 ± 4.13 ^A^	44.27 ± 5.5 ^A^	18.47 ± 1.9 ^B^	22.13 ± 2.7 ^C^	27.38 ± 2.8 ^D^	29.47 ± 3.7 ^D^	0.000	0.000	0.660
INS (mU/L)	237.9 ± 33 ^A^	231.1 ± 28 ^A^	299.2 ± 40 ^B^	265.4 ± 33 ^C^	279.6 ± 39 ^C^	272.9 ± 33 ^C^	0.000	0.027	0.194
NEFA (mmol/L)	0.43 ± 0.1 ^A^	0.43 ± 0.06 ^A^	0.3 ± 0.07 ^B^	0.32 ± 0.1 ^B^	0.37 ± 0.05 ^C^	0.41 ± 0.07 ^D^	0.000	0.217	0.509
BHB (mmol/L)	0.58 ± 0.1 ^A^	0.65 ± 0.1 ^A^	0.32 ± 0.13 ^B^	0.33 ± 0.1 ^C^	0.36 ± 0.12 ^D^	0.34 ± 0.09 ^E^	0.000	0.295	0.245
GLU (mmol/L)	3.12 ± 0.3 ^A^	3.5 ± 0.43 ^B^	2.37 ± 0.18 ^C^	2.69 ± 0.37 ^D^	2.73 ± 0.4 ^D^	2.87 ± 0.4 ^D^	0.000	0.000	0.366
Ca (mmol/L)	2.95 ± 0.12 ^A^	2.68 ± 0.37 ^B^	2.66 ± 0.14 ^B^	2.47 ± 0.31 ^C^	2.66 ± 0.26 ^B^	2.61 ± 0.3 ^B^	0.001	0.002	0.250
P (mmol/L)	2.08 ± 0.24 ^A^	2.04 ± 0.26 ^A^	1.86 ± 0.24 ^B^	1.9 ± 0.2 ^B^	1.95 ± 0.22 ^B^	1.89 ± 0.24 ^B^	0.004	0.665	0.596
Mg (mmol/L)	1.29 ± 0.11 ^A^	1.26 ± 0.1 ^A^	1.22 ± 0.07 ^B^	1.2 ± 0.12 ^B^	1.25 ± 0.11 ^B^	1.17 ± 0.1 ^C^	0.017	0.040	0.375
TPROT (g/L)	64.77 ± 2.6 ^A^	65.4 ± 2.7 ^A^	72.95 ± 3.4 ^B^	73.01 ± 2.6 ^B^	70.17 ± 2.7 ^C^	70.22 ± 3 ^C^	0.000	0.665	0.895
ALB (g/L)	33.34 ± 1.6 ^A^	32.12 ± 1.8 ^A^	40.01 ± 1.8 ^B^	38.08 ± 1.9 ^C^	37.37 ± 1.7 ^D^	36.12 ± 1.3 ^E^	0.000	0.040	0.894
GLB (g/L)	31.44 ± 3.1 ^A^	33.28 ± 2.1 ^A^	32.94 ± 4 ^A^	34.93 ± 3.9 ^B^	32.8 ± 2.9 ^A^	34.1 ± 3.5 ^B^	0.358	0.064	0.948
Urea (mmol/L)	4.58 ± 0.4 ^A^	4.11 ± 0.67 ^A^	5.41 ± 0.53 ^B^	5.15 ± 0.7 ^C^	4.87 ± 0.6 ^A^	4.21 ± 0.6 ^D^	0.000	0.000	0.410
TGC (mmol/L)	0.11 ± 0.03^A^	0.16 ± 0.05 ^B^	0.09 ± 0.03 ^C^	0.13 ± 0.04 ^D^	0.1 ± 0.03 ^A^	0.14 ± 0.05 ^D^	0.111	0.000	0.895
CHOL (mmol/L)	1.30 ± 0.2 ^A^	1.65 ± 0.4 ^B^	0.97 ± 0.2 ^C^	1.3 ± 0.31 ^A^	1.21 ± 0.21 ^A^	1.52 ± 0.4 ^B^	0.000	0.000	0.968
TBIL (µmol/L)	3.15 ± 0.8 ^A^	3.48 ± 0.6 ^A^	4.1 ± 0.83 ^B^	4.24 ± 0.75 ^B^	3.31 ± 0.81 ^A^	3.35 ± 0.54 ^A^	0.000	0.259	0.719
DBIL (µmol/L)	1.24 ± 0.44 ^A^	1.43 ± 0.36 ^A^	1.69 ± 0.6 ^B^	1.73 ± 0.44 ^B^	1.36 ± 0.51 ^C^	1.43 ± 0.4 ^A^	0.003	0.288	0.793
IBIL (µmol/L)	1.91 ± 0.65 ^A^	2.05 ± 0.5 ^A^	2.4 ± 0.45 ^B^	2.51 ± 0.8 ^B^	1.95 ± 0.63 ^A^	1.92 ± 0.36 ^A^	0.001	0.553	0.814
CR (U/L)	88 ± 10.4 ^A^	94.4 ± 6.66 ^A^	110 ± 13 ^B^	106.6 ± 7.6 ^B^	106.3 ± 9.1 ^C^	97.6 ± 10.1 ^B^	0.000	0.332	0.008
AST (U/L)	50.42 ± 9.7 ^A^	53.7 ± 12.8 ^A^	61 ± 10.2 ^B^	63.3 ± 16.5 ^B^	58.12 ± 11 ^C^	58 ± 14.2 ^C^	0.006	0.474	0.857
ALT (U/L)	34.51 ± 2.3 ^A^	35.1 ± 2.07 ^A^	42.65 ± 3.1 ^B^	40.6 ± 2.7 ^B^	38.56 ± 2.5 ^C^	39.05 ± 2.4 ^C^	0.000	0.508	0.061
GGT (U/L)	53.9 ± 13.3 ^A^	57.23 ± 16 ^A^	66 ± 18.6 ^B^	64.9 ± 16.2 ^B^	62.41 ± 17 ^B^	63.31 ± 16 ^B^	0.042	0.748	0.856
LDH (U/L)	129 ± 10.6 ^A^	126.3 ± 17 ^A^	160.5 ± 14 ^B^	148.8 ± 18 ^C^	139.1 ± 16 ^C^	144.1 ± 16 ^C^	0.000	0.276	0.092
ALP (U/L)	118 ± 23.9 ^A^	121.4 ± 14 ^A^	144.8 ± 28 ^B^	141.9 ± 19 ^B^	131.4 ± 24^A^	135.7 ± 20 ^B^	0.000	0.727	0.771
CK (U/L)	165.4 ± 51 ^A^	149.9 ± 11 ^A^	198.4 ± 20 ^B^	193.3 ± 37 ^B^	167.7 ± 14 ^C^	163.2 ± 9.3 ^C^	0.000	0.143	0.676
HOMA	4.77 ± 0.85 ^A^	5.2 ± 1.1 ^A^	4.55 ± 0.75 ^A^	4.62 ± 1.07 ^A^	4.87 ± 0.9 ^A^	5.02 ± 1.02 ^A^	0.162	0.260	0.723
QUICKI	0.31 ± 0.01 ^A^	0.3 ± 0.01 ^A^	0.31 ± 0.01 ^A^	0.31 ± 0.01 ^A^	0.3 ± 0.01 ^A^	0.3 ± 0.01 ^A^	0.130	0.373	0.694
RQUICKI	0.34 ± 0.01 ^A^	0.34 ± 0.01 ^A^	0.37 ± 0.02 ^B^	0.37 ± 0.03 ^B^	0.35 ± 0.01 ^C^	0.34 ± 0.01 ^A^	0.000	0.246	0.906
RQUICKIBHB	0.38 ± 0.01 ^A^	0.36 ± 0.02 ^B^	0.46 ± 0.05 ^C^	0.45 ± 0.05 ^C^	0.42 ± 0.03 ^D^	0.41 ± 0.03 ^E^	0.000	0.158	0.937

* Abbreviations: cortisol (CORT), triiodothyronine(T3), thyroxine (T4), insulin (INS), non-esterified fatty acids (NEFA), beta-hydroxybutyrate (BHB), glucose (GLU), calcium (Ca), inorganic phosphates (P), magnesium (Mg), total protein (TPROT), albumin (ALB), globulin (GLB), urea, triglycerides (TGC), cholesterol (CHOL), total bilirubin (TBIL), direct bilirubin (DBIL), indirect bilirubin (IBIL), creatinine (CR), aspartate aminotransferase (AST), alanine aminotransferase (ALT), gamma-glutamyl transferase (GGT), lactate dehydrogenase (LDH), alkaline phosphatase (ALP) and creatine kinase (CK), homeostasis model assessment (HOMA), quantitative insulin sensitivity check index (QUICKI), revised quantitative insulin sensitivity check index (RQUICKI) and its modified version (RQUICKIBHB). ^A,B,C,D,E^—different superscripts indicate statistically significant differences between subgroups.

**Table 3:** Correlation between examined blood parameters and body temperature measured rectally and by thermography during the entire experimental period.

	Rectal Temperature (RT)	Nose Temperature (NT)	Eye Temperature (ET)	Leg Temperature (LT)	Abdomen Temperature (AT)
COR ^1^	−0.109	−0.005	0.040	0.448 **	0.474 **
T3	−0.082	−0.196	−0.247 *	−0.698 **	−0.723 **
T4	−0.091	−0.229 *	−0.224 *	−0.760 **	−0.776 **
INS	0.096	0.153	0.099	0.445 **	0.427 **
NEFA	−0.104	−0.143	−0.226 *	−0.451 **	−0.468 **
BHB	−0.046	−0.055	−0.107	−0.627 **	−0.632 **
GLU	−0.026	−0.208 *	−0.259 **	−0.516 **	−0.547 **
Ca	−0.260 **	−0.195	−0.254 *	−0.447 **	−0.416 **
P	−0.028	−0.105	−0.156	−0.269 **	−0.216 *
Mg	−0.051	−0.022	−0.072	−0.315 **	−0.259 **
TPROT	0.005	0.152	0.123	0.651 **	0.656 **
ALB	0.037	0.153	0.172	0.449 **	0.492 **
GLB	−0.032	−0.003	−0.049	0.185	0.149
Urea	−0.051	0.055	0.033	0.319 **	0.321 **
TGC	0.225*	0.125	0.080	−0.058	−0.024
CHOL	0.105	0.010	−0.062	−0.233 *	−0.225 *
TBIL	0.074	0.331 **	0.140	0.321 **	0.372 **
DBIL	0.183	0.328 **	0.092	0.268 **	0.325 **
IBIL	−0.045	0.183	0.114	0.218 *	0.242 *
CR	0.196	0.317 **	0.183	0.550 **	0.572 **
AST	0.036	0.149	0.178	0.232 *	0.324 **
ALT	0.111	0.355 **	0.269 **	0.666 **	0.693 **
GGT	0.103	0.090	0.145	0.283 **	0.292 **
LDH	0.101	0.214 *	0.212 *	0.471 **	0.554 **
ALP	−0.041	0.044	0.207 *	0.366 **	0.350 **
CK	0.126	0.305 **	0.177	0.290 **	0.384 **
HOMA	0.048	−0.072	−0.170	−0.089	−0.134
QUICKI	−0.029	0.089	0.160	0.105	0.139
RQUICKI	0.078	0.183	0.270 **	0.386 **	0.403 **
RQUICKIBHB	0.126	0.183	0.277 **	0.625 **	0.639 **

^1^—abbreviations: see Table 2. **—correlation is significant at *p* < 0.01 level. *—correlation is significant at *p* < 0.05 level.

## Data Availability

The data presented in this study are available in the article.
